# Dopaminergic Receptors and Tyrosine Hydroxylase Expression in Peripheral Blood Mononuclear Cells: A Distinct Pattern in Central Obesity

**DOI:** 10.1371/journal.pone.0147483

**Published:** 2016-01-25

**Authors:** Fernanda Leite, Margarida Lima, Franca Marino, Marco Cosentino, Laura Ribeiro

**Affiliations:** 1 Department of Biochemistry, Faculty of Medicine, University of Porto, Porto, Portugal; 2 Department of Clinical Haematology, Centro Hospitalar of Porto, Porto, Portugal; 3 Unit for Multidisciplinary Research in Biomedicine, Abel Salazar Institute of Biomedical Sciences, University of Porto- UMIB/ICBAS/UP, Porto, Portugal; 4 Center of Research in Medical Pharmacology, University of Insubria, Varese, Italy; 5 Instituto de Investigação e Inovação em Saúde (I3S), University of Porto, Porto, Portugal; 6 Department of Medical Education and Simulation, Faculty of Medicine, University of Porto, Porto, Portugal; Hudson Institute, AUSTRALIA

## Abstract

**Background:**

Dopamine (DA) may be involved in central obesity (CO), an inflammatory condition, through its role in the central nervous system and in periphery, where it may affect immune cell function through five different DA receptors (DR). Whether dopaminergic pathways in peripheral immune cells are implicated in the inflammatory condition linked to CO is however unknown.

**Methods:**

In a cohort of blood donors with and without CO, categorized by waist circumference (WC) (CO: WC ≥0.80 m in women and ≥0.94 m in men), we studied the expression of DR and tyrosine hydroxylase (TH), the rate-limiting enzyme in the synthesis of DA, in peripheral blood mononuclear cells (PBMCs) and their relation with anthropometric and metabolic/endocrine and inflammatory parameters. DR D_1-5_ and TH expression was assessed by semi quantitative real-time PCR. As inflammatory markers we investigated the immunophenotype of monocyte subsets by flow cytometry, staining for CD14, CD16, CD11b and CD36.

**Results:**

CO individuals showed higher plasma levels of leptin and higher inflammatory pattern of monocytes compared with non-CO. PBMC expression of DR D_2_, DR D_4_ and DR D_5_ as well as of TH were lower in CO in comparison with non-CO. DR D_2_, and DR D_5_ expression correlated with lower WC and weight, and with lower inflammatory pattern of monocytes, and TH expression correlated with lower WC. DR D_4_ expression correlated with lower plasma levels of glycosylated hemoglobin, and DR D_2_ expression correlated with lower CO.

**Conclusions:**

Results show that CO is associated with peripheral inflammation and downregulation of dopaminergic pathways in PBMCs, possibly suggesting DR expressed on immune cells as pharmacological targets in obesity for better metabolic outcome.

## Introduction

The modern obesity epidemic is a major public health issue, with the principal cause of morbidity due to metabolic dysfunction such as insulin resistance, type 2 diabetes, dyslipidemia and cardiovascular disease [1]. Although it is widely accepted that a state of chronic low grade inflammation is responsible for the metabolic dysfunction of obesity, its precise etiology is not completely characterized.

The expression of common receptors and signaling networks in immune cells and adipocytes is the basis of the immunological metabolic cross-talk that explains the inflammatory comorbidities of obesity [2]. On the one hand, immune cells play a central role in adipose tissue biology and, on the other hand, adipocytes have been recently proposed as “immune cells” since they express, cytokines, chemokines, multiple receptors and cell molecules involved in the immune response [3]. Visceral or central obesity (CO) is indeed regarded as an inflammatory disease [1], and recent research has been focused on the study of peripheral blood mononuclear cells (PBMCs) in obesity. Indeed, PBMCs are exposed to systemic factors, such nutrients and inflammatory molecules [4], and may constitute potential biomarkers of early homeostatic energy imbalance, and reducing inflammation could be useful in preventing the occurrence of obesity and its consequences [5].

Dopamine (DA), a classical brain neurotransmitter involved in various vital central nervous system functions, including feeding, reward and cognition, has been reported to modulate peripheral immune function [6–8]. Indeed, DA-induced immunomodulation is currently the focus of intense experimental research and dopaminergic pathways are increasingly considered a target for drug development in immune disease [9]. Immune cells themselves produce endogenous DA, and possibly uptake DA from other sources, using this transmitter as an autocrine/paracrine mediator [10–12]. The first and rate limiting step of DA biosynthesis is the hydroxylation of the L-tyrosine to L-DOPA via tyrosine hydroxylase (TH). Once released, DA can bind to five distinct G protein-coupled receptors [13]. Dopaminergic receptors (DR) are grouped into two families according to their pharmacological profile and main second messenger coupling: the D_1_-like (D_1_ and D_5_) which activate adenylate cyclase and the D_2_-like (D_2_, D_3_ and D_4_) which inhibit adenylate cyclase [13].

While several lines of evidence support the involvement of DA in obesity through its role in the central nervous system (CNS) [14–16] no information exists so far regarding dopaminergic pathways in peripheral immune cells of obese subjects. We therefore decided to study the expression of DR and TH in PBMCs from a cohort of blood donors and their relation with anthropometric, metabolic and inflammatory parameters in central obesity.

## Methods

### Ethics statement

This work was approved by the Ethical Committee of Centro Hospitalar of Porto (Porto, Portugal). All participants signed a written informed consent as described in the consent procedure of the study protocol approved by the Ethics committee. The clinical study is registered at the local research department with the identifier 072/09 (047-DEFI/065-CES).

### Study Population

Thirty blood donors from the blood bank of Clinical Haematology department of Centro Hospitalar of Porto were enrolled in the study. The individuals followed the selection criteria for blood donation and were not under any medicines for at least one month before enrolment. Systolic blood pressure (SBP) and diastolic blood pressure (DBP) were measured twice and the mean values were used in the analyses; readings were taken on the right arm, with subjects in the supine position after they had rested for 5 min.

### Anthropometrics

Height (in m) was based on an identification document and confirmed by medical record. Body weight was measured to the nearest 100 g on electronic weight scales. Body mass index (BMI) was calculated by dividing weight by height squared and expressed in kg x m^-2^ and categorized as described before [17]. Waist circumference (WC) was measured with a flexible tape at the level midway between the lowest rib and the iliac crest. CO was defined following International Diabetes Federation criteria by using WC (≥0.80 m for women and ≥0.94 m for men) [18].

### Biochemical analysis

Blood samples were taken under standardized conditions. Fasting plasma glucose, triacylglycerol (TAG), total cholesterol (TC), high-density lipoprotein-cholesterol (HDL-C), low-density lipoprotein-cholesterokg.m-2l (LDL-C), very low-density lipoprotein-cholesterol (VLDL-C), glycosylated hemoglobin (HbA1c) were measured using standard techniques. Plasma catecholamines (CA) [adrenaline (AD) and noradrenaline (NA)] were assayed by high pressure liquid chromatography with electrochemical detection (HPLC-ED) (CROMSYSTEMS). Leptin was measured in serum by solid phase two-site enzyme immunoassay (Merecodia Leptin ELISA) and high-sensitivity C-reactive protein (hsCRP) by nephelometry (CardioPhase hsCRP–BnProSpec Siemens).

### Flow Cytometry assay of monocytes

Monocytes were analyzed in whole blood samples by means of flow cytometry following a previously described technique [19]. The enumeration and the imunophenotypic analysis of monocytes were performed in fresh EDTA-K3 anti-coagulated blood samples. Immunophenotypic studies were done using a whole blood stain-lyse-and-then-wash method and a direct immunofluorescence technique using the following four-color panel of monoclonal antibodies: mouse anti-human CD36 conjugated with FITC (clone FA6.152, IgG1), mouse anti-human CD16 conjugated with PE (clone 3G8, IgG1), mouse anti-human CD14 conjugated with PE-Cy5 (IgG2a, clone RMO52) all obtained from Beckman Coulter (catalogue numbers IM0766U, IM1238U and IM2640U, respectively), and mouse anti-human CD11b conjugated with APC (IgG2a, clone D12) obtained from Becton Dickinson (BD) (catalogue number 333143).

Data acquisition was carried out on a FACS-Calibur flow cytometer (BD), using the Cell Quest software program (BD). Information on a minimum of 2x10^5^ events was acquired for each staining and stored as list mode data. For data analysis, the Paint-a-Gate Pro software program (BD) was used. Monocytes were quantified based on the CD14 expression, while CD16 was used to differentiate classical (CD16-) and non-classical (CD16+) monocyte populations. The median fluorescence intensity (MFI) of CD14, CD36 and CD11b was assessed in each subset and expressed as fluorescence arbitrary units (AU). The forward scatter (FSC) and side scatter (SSC) of the cell subsets were also measured. In order to overcome inter-individual variations, we calculated the ratio for each parameter between non-classical CD14+CD16+ and classical CD14+CD16- monocytes in each individual.

### Real time PCR analysis for the expression of DR and TH in PBMCs

Peripheral blood mononuclear cells were isolated by density gradient centrifugation (Ficoll method) and DR and TH mRNA were assayed by real time PCR as previously described [20]. Briefly, total RNA was extracted from PBMCs by PerfectPure RNA Cell & Tissue kit (5Prime), and the amount of extracted RNA was estimated by spectrophotometry at 260 nm. Total RNA was then reverse transcribed using the High-capacity cDNA Archive Kit (Applied Biosystems, Foster City, USA), according to the manufacturer’s instructions. Real-time PCR was performed with an ABI prism 7000 apparatus (Applied Biosystems) using the Assay on demand kits for the genes of interest (Applied Biosystems), according to the manufacturer’s instructions. Gene sequence data were obtained from the Reference Sequence collection (RefSeq; www.ncbi.nlm.nih.gov/projects/RefSeq). The thermal profile for each gene was: stage 1, 2 min at 50°C; stage 2, 10 min at 95°C; stage 3, 40 cycles including 15 s at 95°C and 1 min at 60°C. Further details about real-time PCR conditions are shown in Table 1. Linearity of real-time PCR assays were tested by constructing standard curves by use of serial 2-fold dilutions of a standard calibrator cDNA and regression coefficients (r^2^) were always >0.900 (data not shown). Relative expression was determined by normalization to 18S rRNA (housekeeping gene) by means of AB Prism 7000 SDS software. S1 Fig shows HKG levels (expressed as Ct) for the different genes, and also the comparison between the two groups of subjects. No difference is statistically significant and the range of the individual values is very narrow (about one cycle) and clearly superimposed between groups and throughout genes. In addition, HKG minor variations are not influential and likely due to small variations in the amount of cDNA pipetted for the PCR analysis. Gene expression levels in a given sample were represented as 2^-Δ**Ct**^ where ΔCt = [Ct (gene)–Ct (18S rRNA)]. The ratio (R) was calculated for DR and TH mRNA expression between individuals with and without CO. R<0.5 was conventionally considered as underexpression and R>2.0 as overexpression.

**Table 1:** Real-Time PCR gene expression. Data are from RefSeq—NCBI Reference Sequence Database (http://www.ncbi.nlm.nih.gov/refseq/).

Gene	UniGene ID	Interrogated Sequence	Translated Protein	Exon Boundary	Assay Location	Amplicon Length
TH	Hs.435609	NM_199292.2	NP_954986.2	3–4	424–422	63
DRD_1_	Hs.2624	NM_000794.3	NP_000785.1	1–2	462–1620	110
DRD_2_	Hs.73893	NM_000795.3	NP_000786.1	2–3	524	64
DRD_3_	Hs.121478	NM_033663.3	NP_387512.3	3–4	809–725	73
DRD_4_	Hs.99922	NM_000797.3	NP_000788.2	1–2	285–283	99
DRD_5_	Hs.380681	NM_000798.4	NP_000789.1	1–1	1092–744	88
18SrRNA	X03205.1	N/A	N/A	N/A	N/A	187

Abbreviations: TH, tyrosine hydroxylase; DRD_1_, dopaminergic receptor type1; DRD_2_, dopaminergic receptor type 2; DRD_3_, dopaminergic receptor type 3; DRD_4_, dopaminergic receptor type 4; DRD_5_, dopaminergic receptor type 5.

All the parameters were assessed in 30 (17 with and 13 without CO) individuals, except leptin and CD11b, assessed in 21 (11 with and 10 without CO) and 13 (8 with and 5 without CO), respectively.

### Statistical Analysis

The modified Kolmogorov-Smirnov test with the correction of Lilliefors was used to evaluate the fit of the data to a normal distribution. Variables were summarized using relative and absolute frequencies or means±standard error of the mean (SEM), as appropriate. Non-normally distributed data were summarized as median, 25th and 75th percentiles. To compare the quantitative independent variables we used bivariate ANOVA or Mann-Whitney tests for normally and non-normally distributed data, respectively. The Pearson Chi-Square test was used to compare qualitative independent variables. Correlations were assessed by non-parametric Spearman rank analysis. Only those correlations with significance lower or equal to 0.01 were considered. The strength of association between variables was estimated by odds ratio (OR) and their respective 95% confidence interval (CI) using multiple logistic regression. Variables that in the univariate analysis showed statistical significance below 10% (p <0.10) were included in the logistic regression model. Data analysis was performed with the version 22.0 software Predictive Analytics Software (PASW Statistics Software).

## Results

### Characteristics of the study population

In this cohort of blood donors the prevalence of central obesity was 57.7%, and the mean BMI was 30±1.5 and 24±0.7 kg.m-2, respectively in individuals with and without CO (Table 2). There were no differences between subjects without and with CO, with the exception of plasma leptin which was higher in CO.

**Table 2:** Characteristics of the participants (n = 30) and comparison between groups defined by Waist Circumference.

		Overall			Waist Circumference		
Parameter	Reference values (range)	mean± SEM/*	*F*	*df*	*No CO* (n = 13)	*CO* (n = 17)	*P*
**Age (years)**		41±2	0.795	1, 28	39±3	42±2	0.380
**Weight (Kg)**		76±3	3.302	1, 28	70±2	80±4	0.080
**Height (m)**		1.67±0.02	5.780	1, 28	1.71±0.01	1.64±0.02	0.023
**BMI (kg.m**^**-2**^**)**		27.2±1.0	10.448	1, 28	24±0.7	30±1.5	0.003
**WC (m)**		0.95±0.03	13.492	1, 28	0.86±0.02	1.02±0.04	0.001
**SBP (mmHg)**	<130	134±3	0.120	1, 28	133±4	135±5	0.732
**DBP (mmHg)**	<85	79±2	0.362	1, 28	77±2	80±3	0.552
**Glycemia (mg/dL)**	70–105	85±1	0.200	1, 28	86±1	84±2	0.659
**HgA1c (%)**	3.8–5.6	5.2±0.06	2.070	1, 28	5.1±0.08	5.3±0.07	0.161
**TC (mg/dL)**	0–200	188±7	0.807	1, 28	181±10	193±9	0.377
**LDL-C (mg/dL)**	0–130	117±6	0.084	1, 28	115±9	118±8	0.774
**HDL-C (mg/dL)**	35–55	51±3	0.080	1, 28	50±4	52±4	0.780
**VLDL-C (mg/dL)**	3–56	20±2	2.901	1, 28	16±2	23±3	0.100
**TAG (mg/dL)**	40–160	99±10	2.855	1, 28	80±9	113±16	0.102
**NA (pmoL/L)**	709–4019	879±131	0.444	1, 28	778±185	956±185	0.511
**AD (pmoL/L)**	<328	167±17	0.172	1, 28	175±31	160±18	0.682
**Cortisol (μg/dL)**	6.2–19.4	15±0.8	0.439	1, 28	16±1	15±1	0.513
Leptin (ng/mL)**	2.0–5.6	0.9±0.3	6.779	1, 19	0.138±0.047	1.626±0.542	0.017
**Leucocytes (cells/μL)**	4500–13000	6403±280	1.823	1, 28	5977±483	6729±319	0.188
**Monocytes (cells/μL)**	400–500	446±31	1.361	1, 28	487±55	415±34	0.253
**Lymphocytes (cells/μL)**	1000–4800	1951±96	3.729	1, 28	1748±123	2106±131	0.064

Abbreviations: BMI, Body Mass Index; WC, waist circumference; SBP, systolic blood pressure; DBP, diastolic blood pressure; HbA1c, glycosylated hemoglobin; TC, total cholesterol; LDL-C, low-density lipoprotein cholesterol; HDL-C, high-density lipoprotein-cholesterol; VLDL-C, very low-density lipoprotein-cholesterol; TAG, triacylglycerol; NA, noradrenaline; AD, adrenaline; F Snedcor’s distribution; df default freedom; p level of significance. Data are presented as mean ± standard error of the mean (SEM). Differences between groups were analyzed by ANOVA.

*Data presented as median, 25^th^ and 75^th^ percentiles and statistical analysis performed with Mann- Whitney U test for the differences between the 2 groups.

**Leptin was assessed in 21 individuals (11 with and 10 without CO).

Plasma CA levels were similar between WC established groups, but in CO AD correlated with leptin (*r* = 0.860; *P* = 0.001). In the whole population, total monocytes were 446±31 cells/μL and no differences were noticed between subjects with and without CO (415±34 vs 487±55, respectively; p = 0.253).

Total monocyte count as well as CD16+ and CD16- monocytes were not different between subjects with and without CO (Tables 2 and 3) however, in comparison to monocytes from subjects without CO, cells from subjects with CO showed lower SSC, CD14, CD11b, and CD36 ratios (Table 3), defining a higher inflammatory phenotype for pro-inflammatory monocytes.

**Table 3:** Immunophenotypic characteristics of CD16+ and CD16- monocytes in overall participants and categorized by waist circumference.

Parameter	Overall	*p	Without CO	With CO	p
**cells/μL**					
CD16+	54±12		57±21	53±13	
CD16-	392±25		430±43	362±28	
CD16+/CD16-	0.14±0.02	<0.001	0.12±0.03	0.15±0.03	ns
**Cells %**					
CD16+	11.4±1.4		10±2	12±2	
CD16-	88.6±1.4		90±2	88±2	
CD16+/CD16-	0.14±0.02	<0.001	0.13±0.03	0.15±0.03	ns
**FSC**					
CD16+	557±11		546±18	560±13	
CD16-	558±11		548±17	565±13	
CD16+/CD16-	1±0.005	ns	1.00±0.01	1.00±0.01	ns
**SSC**					
CD16+	418±10		435±17	405±11	
CD16-	486±8		488±13	483±10	
CD16+/CD16-	0.86±0.01	<0.001	0.89±0.02	0.84±0.01	0.022
**CD14**					
CD16+	875±109		893±163	860±151	
CD16-	2340±322		1874±353	2561±476	
CD16+/CD16-	0.41±0.03	<0.001	0.51±0.05	0.34±0.03	0.009
**CD36**					
CD16+	54±12		332±59	282±28	
CD16-	392±25		671±78	781±45	
CD16+/CD16-	0.43±0.03	<0.001	0.51±0.05	0.38±0.03	0.023
CD11b**					
CD16+	11.4±1.4		90±35	115±35	
CD16-	88.6±1.4		143±54	405±174	
CD16+/CD16-	0.49±0.05	0.050	0.64±0.05	0.40±0.06	0.015

Abbreviations: CO, central obesity; FSC, forward scatter; SSC, side scatter; ns, not significant. Values of CD14, CD36 and CD11b expressed as fluorescence arbitrary units (AU). Data are presented as mean ± standard error of the mean (SEM). Differences between groups analyzed by ANOVA p, level of significance; p* significance between CD16+ and CD16- monocytes subsets.

**CD11b was assessed in 13 individuals (8 with and 5 without CO).

### Expression of DR and TH in PBMCs

**Fig 1:**
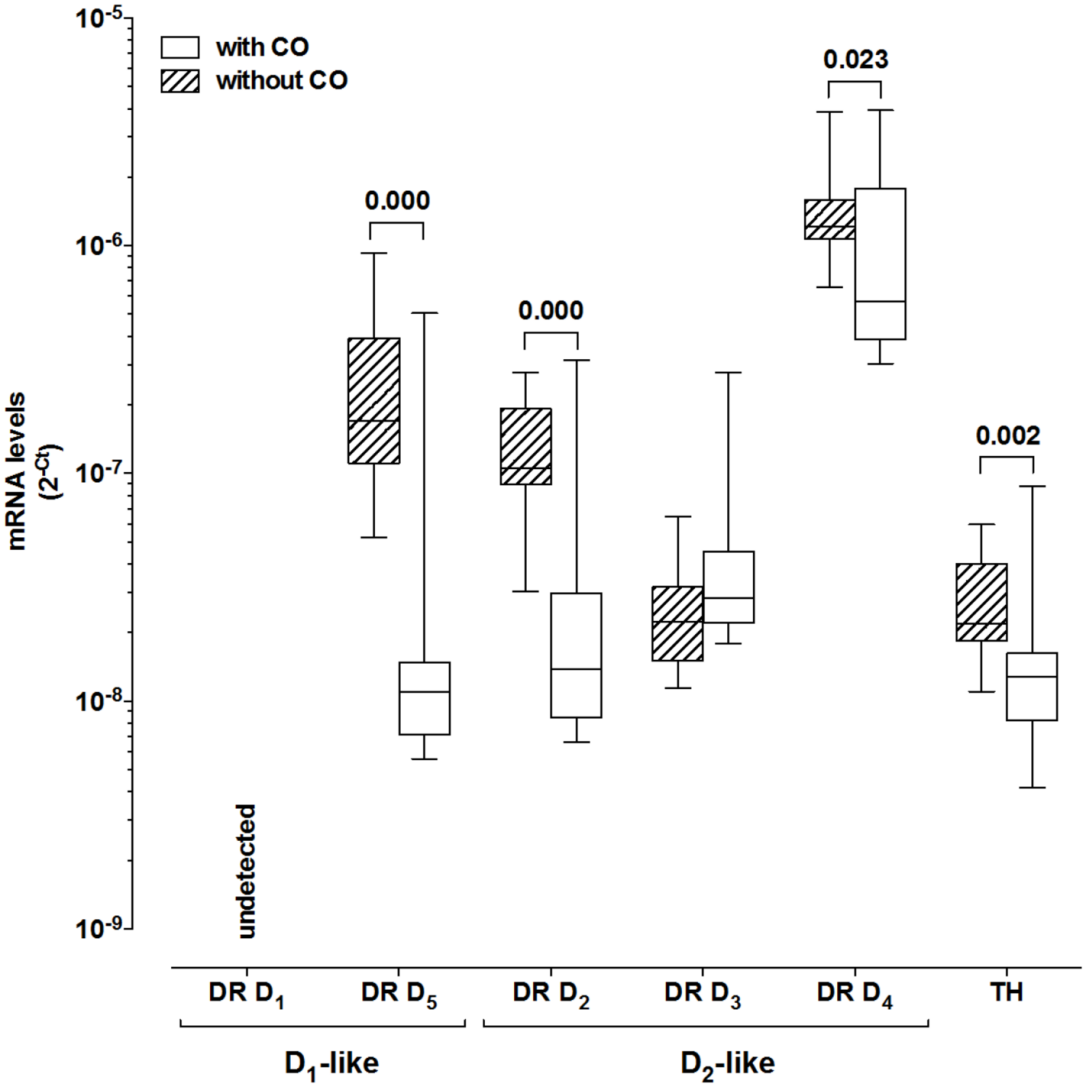
Comparison of the expression of Dopaminergic receptors in Peripheral Blood Mononuclear Cells between Groups with and without Central Obesity.

Compared to PBMCs from subjects without CO, cells from individuals with CO expressed lower D1-like DR D5 (R = 0.29) and D2-like DR D2 (R = 0.13) and D_4_ (R = 0.47), as well as lower TH mRNA (R = 0.58) (Fig 1). In centrally obese individuals, the comparison between obese and non-obese BMI defined did not show differences in mRNA levels of TH and DR in PBMCs (S1 Table).

Boxes indicate medians with 25th–75th percentiles and whiskers indicate minimum and maximum values. Mann-Whitney test used for comparison between the two groups. Abbreviations: Tyrosine hydroxylase (TH), Central Obesity (CO), DRD1 dopaminergic receptor type1, DRD2 dopaminergic receptor type 2, DRD3 dopaminergic receptor type 3, DRD4 dopaminergic receptor type 4, DRD5 dopaminergic receptor type 5, Significant differences are indicated (P value).

### Correlation between DR and TH mRNA expression in PBMCs and anthropometric, metabolic/endocrine and inflammatory markers

As shown in Table 4, in the whole cohort, the expression of DR D_2_ and DR D_5_ mRNA in PBMCs was negatively correlated with weight, BMI, WC and leptin levels, and positively correlated with CD11b ratio in monocytes. Moreover, DR D_4_ mRNA was negatively correlated with HbA1c and TH mRNA was correlated with lower WC and leptin levels.

**Table 4:** Correlations between dopaminergic receptors and tyrosine hydroxylase expression and anthropometric, metabolic/endocrine and inflammatory parameters.

	TH		DRD_2_		DRD_3_		DRD_4_		DRD_5_	
	*r*	*P*	*r*	*P*	*r*	*P*	*r*	*P*	*r*	*P*
Weight	ns		-0.502	0.005	ns		ns		-0.483	0.007
BMI	ns		-0.577	0.001	ns		ns		-0.557	0.001
WC	-0.529	0.003	-0.681	<0.001	ns		ns		-0.621	<0.001
HbA1c	ns		ns		ns		-0.605	<0.001	ns	
Leptin	-0.555	0.009	-0.739	<0.001	ns		ns		-0.618	0.003
Ratio CD11b	ns		0.927	<0.001	ns		ns		0.748	0.003

Abbreviations: *TH* tyrosine hydroxylase, *DRD*_*2*_ dopaminergic receptor type *2*, *DRD*_*3*_ dopaminergic receptor type *3*, *DRD*_*4*_ dopaminergic receptor type *4*, *DRD*_*5*_ dopaminergic receptor type *5*, *BMI*, Body mass index; *WC* waist circumference; *HbA1c* glycosylated hemoglobin; *Ratio CD11b*, ratio between the expression of CD11b in CD16+ monocytes and the expression of CD11b in CD16- monocytes and the median fluorescence intensity of CD11b was assessed in each subset and expressed as fluorescence arbitrary units; *r c*orrelation coefficients, calculated by Spearman’s rho test; *P* level of significance; *ns* not significant if *P>0*.*01*.

In the model of CO, the logistic regression analysis showed a lower association for the development of central obesity for DR D2 mRNA expression ≥0.0000000455 (odds ratio [OR] 0.018 confidence interval [CI] 95% 0002–0.195).

All data are included in the supporting information (S1 Dataset).

## Discussion

This is the first work studying the expression of DR and TH in peripheral immune cells in obesity. Our findings show that CO is associated with inflammation, as shown by higher plasma levels of leptin and a more inflammatory pattern of non-classical monocytes, and that in CO PBMCs exhibit a distinct pattern of DR and TH expression. In particular: (i) PBMCs from subjects with CO show a reduction of the expression of DR D_2_, DR D_4_ and DR D_5_ as well as lower levels of TH mRNA in comparison to cells from the non-obese group; (ii) DR D_2_ and DR D_5_ expression in PBMCs strongly correlates with lower weight, BMI and WC, lower plasma levels of leptin and with a lower inflammatory pattern, while DR D_4_ mRNA correlates with less HbA1c and TH mRNA correlates with lower WC and leptin levels; (iii) the expression of DR D_2_ may have a protective role against the presence of CO.

The relationship between DA and obesity has been subject of extensive research in the nervous system [14–16, 21]. Scientific evidence has established an association between obesity and hyposensitivity of dopaminergic systems, both within the CNS [22] and in peripheral tissues [23]. While DA brain functions are well known, its peripheral role is an emerging subject of study. An association between the intake of a high fat diet and alterations in brain DA levels [24, 25] has been already described, and Wang *et al*. (2001) reported that in obese DR D_2_ availability correlated negatively with BMI [22].

Till now, knowledge about the role of DA in peripheral tissues during obesity has been limited to its influence on pancreatic β cells regulating insulin release [26] and to the modulation of insulin effects on adipocytes [21]. Indeed, Borcherding et al (2011) suggested a regulatory role of peripheral DA in adipose tissue functions as human adipocytes cell lines express DR [27].

In our study, we found decreased DR D_2_, D_4_ and D_5_ as well as TH mRNA levels in PBMCs from subjects with CO. Decreased expression of DR D_2_ in PBMCs has been already reported in other inflammatory immune mediated diseases such as Crohn’s disease [28] and systemic lupus erythematosus (SLE) [29]. Indeed, administration of DR D_2_ agonists reduced disease activity in patients with rheumatoid arthritis [30, 31] and decreased serum immunoglobulin and anti-DNA antibody levels in SLE patients [30]. Single-nucleotide polymorphisms of DR D_2_, associated to lower expression and function, increase inflammation in human renal proximal tubule cells [32]. In human lymphocytes, DR D_2_ agonists increase the secretion of anti-inflammatory cytokines [33]. As regards DR D_5_, their reduction in PBMCs has been previously reported in patients suffering from multiple sclerosis [34, 35], an immune-mediated inflammatory disease in which DR D_5_ expression in PBMCs has been also suggested to predict the therapeutic response to immunomodulating treatment with interferon- β [35].

In our study, PBMCs expression of DR D_2_ and D_5_ (and to a lesser extent also of D_4_ and TH) were shown to be associated with lower weight, with better metabolic/endocrine parameters and with a less inflammatory pattern. Moreover, logistic regression analysis suggests that DR D_2_ expression has a protective role for the existence of CO.

The correlations between the expression of DR and TH in PBMCs and more favorable metabolic/endocrine and inflammatory parameters suggest a possible role for dopaminergic pathways in the crosstalk between immunity and metabolism. Indeed, adipose tissue express DR and DA regulates adipose tissue functions [27]. So far, DA has been shown to have an inhibitory effect on leptin release from human adipocytes through D_1_-like DR receptors [27] and to modify the secretion of insulin in peripheral tissues [21]. Yu et al. (2014) described a negative effect through DR D_4_ activation on insulin action via decreasing insulin receptor expression [36]. Peripheral blockade of DR could therefore result in increased insulin release and sensitivity, thus promoting adipogenesis, weight gain, insulin resistance, and ultimately type 2 diabetes [21], adverse metabolic effects also described for neuroleptics [37]. DA inhibitory effects on the release and action of insulin may explain the inverse correlation between DR D_4_ expression in PBMCs and HbA1c plasma levels found in the present work. The inhibitory effect of DA on leptin release mediated by DR is in agreement with the relationship found between DR expression and leptin, considering PBMCs as early sensors for metabolic dysfunction. The major sources of DA in peripheral blood are neuronal fibers, adrenal medulla and neuroendocrine cells [38], and although the type of food ingested could affect blood DA levels [21, 39, 40] and putatively the expression and function of DR, in our study blood samples were collected in a fasted state.

Our work has some limitations. First, we did not analyze DR at protein level. Even though, Araki et al (2007) showed that DR mRNA and membrane-associated protein changes correspond with each other; moreover the same authors demonstrated that DR mRNA expression or receptor protein are valid surrogates for receptor function [41] and our group previously showed that DR responsiveness in human lymphocytes is better predicted by mRNA rather than membrane receptor expression [12].

Secondly, we do not know if the different pattern of DR and TH expression on PBMCs associated with CO is a cause or consequence of inflammatory obesity. This open question should be answered prospectively with further studies addressing the effect of weight loss on DR expression in peripheral immune cells. Finally, DR expression was assessed in PBMCs as a whole, and future studies should address the possible differential expression on distinct mononuclear cell subsets (e.g. lymphocytes vs monocytes, T helper (TH) 1 vs Th2, regulatory T cells vs Th17).

## Conclusions

To our knowledge, this is the first work studying the expression of DR and TH in peripheral immune cells in obesity. CO is associated with under expression of DR D_2_, DR D_4_ and DR D_5_, lower levels of TH mRNA in PBMCs and with a higher innate inflammatory pattern. The expression of DR correlated with lower weight, with better metabolic profile and lower inflammatory pattern. The expression of DR D_2_ showed a protective role for the development of CO. Results thus indicate that CO is associated with peripheral inflammation and downregulation of dopaminergic pathways in PBMCs, and warrant further research to establish whether DR expressed on immune cells could represent pharmacological targets in obesity for better metabolic outcome.

## Supporting Information

S1 Dataset Main study data. (XLSX)

S1 Fig Comparison of HKG levels (expressed as Ct) for the different DRD and TH genes between subjects with and without central obesity (CO). (DOCX)

S1 Table Comparison in central obesity (CO) of TH and DR expression in PBMCs between individuals with and without obesity BMI defined. (DOCX)
