# Supplementary material for: Dopaminergic Receptors and Tyrosine Hydroxylase Expression in Peripheral Blood Mononuclear Cells: A Distinct Pattern in Central Obesity
Source: PLoS One. 2016 Jan 25;11(1):e0147483. doi: 10.1371/journal.pone.0147483 (PMC4726756; doi:10.1371/journal.pone.0147483)
Supplement: S1 Fig — (DOCX) [file pone.0147483.s002.docx]

S2 Figure 1. Comparison of HKG levels (expressed as Ct) for the different DRD and TH genes between subjects with and without central obesity (CO). No difference is statistically significant and the range of the individual values is very narrow (about one cycle) and clearly superimposed between groups and throughout genes. In addition, HKG minor variations are not influential and likely due to small variations in the amount of cDNA pipetted for the PCR analysis.
