# Supplementary material for: Dopaminergic Receptors and Tyrosine Hydroxylase Expression in Peripheral Blood Mononuclear Cells: A Distinct Pattern in Central Obesity
Source: PLoS One. 2016 Jan 25;11(1):e0147483. doi: 10.1371/journal.pone.0147483 (PMC4726756; doi:10.1371/journal.pone.0147483)
Supplement: S1 Table — (DOCX) [file pone.0147483.s003.docx]

| S3Table.1 Mean Comparison in CO of the expression of TH and DR in PBMCs between individuals with and without obesity BMI defined. | | | | | |
| --- | --- | --- | --- | --- | --- |
|  | | | | | |
| factor | F | *df* | Mean ± S.E.M. | | p |
|  |  |  | With Obesity (n=10) | Without Obesity (n=7) |  |
| TH | 1.240 | 1, 15 | 1.41x10^-8^ ± 3.27x10^-9^ | 2.51x10^-8^ ± 1.10x10^-8^ | 0.283 |
|  |  |  |  |  |  |
| DRD_2_ | 3.005 | 1, 15 | 1.89x10^-8^ ± 5.49x10^-9^ | 9.20 x10^-8^ ± 5.05 x10^-8^ | 0.103 |
|  |  |  |  |  |  |
| DRD_3_ | 0.605 | 1, 15 | 5.61 x10^-8^ ± 2.49 x10^-8^ | 3.26 x10^-8^ ± 4.67 x10^-9^ | 0.449 |
|  |  |  |  |  |  |
| DRD_4_ | 0.085 | 1, 15 | 1.26 x10^-6^ ± 4.46 x10^-7^ | 1.08x10^-6^ ± 3.89x10^-7^ | 0.775 |
|  |  |  |  |  |  |
| DRD_5_ | 2.140 | 1, 15 | 2.63x10^-8^± 1.78 x10^-8^ | 1.22x10^-7^ ± 7.51x10^-9^ | 0.164 |
| *Abbreviations:CO*, central obesity; *BMI*, body mass index; *TH* tyrosine hydroxylase; *DRD_2-5_*_,_ dopaminergic receptors D_2-5_;*PBMCs*, peripheral blood mononuclear cells; *F,* Snedcor’s distribution; *df,* default freedom; *p,* level of significance. Variables were summarized using means ± standard error of the mean (*SEM*); | | | | | |
